# Population Pharmacokinetics of Polymyxin B and Dosage Optimization in Renal Transplant Patients

**DOI:** 10.3389/fphar.2021.727170

**Published:** 2021-08-25

**Authors:** Ying Li, Yang Deng, Zhen-Yu Zhu, Yi-Ping Liu, Ping Xu, Xin Li, Yue-Liang Xie, Heng-Chang Yao, Liu Yang, Bi-Kui Zhang, Yan-Gang Zhou

**Affiliations:** ^1^ Department of Pharmacy, The Second Xiangya Hospital, Central South University, Changsha, China; ^2^ Institute of Clinical Pharmacy, Central South University, Changsha, China; ^3^ School of Pharmaceutical Sciences, Central South University, Changsha, China; ^4^ Department of Pharmacy, Third Hospital of Changsha, Changsha, China; ^5^ Department of Pharmacy, The Third Xiangya Hospital of Central South University, Changsha, China; ^6^ Department of Urological Organ Transplantation, The Second Xiangya Hospital, Central South University, Changsha, China; ^7^ Hubei Institute of Land Surveying and Mapping, Wuhan, China

**Keywords:** polymyxin B, PPK (population pharmacokinetics), renal transplant, neurotoxicity, dosing regimens

## Abstract

Currently, polymyxin B has been widely used in the treatment of multidrug-resistant Gram-negative pathogen infections. Due to the limited pharmacokinetic/pharmacodynamic data, the optimal dosage regimen for the recently proposed therapeutic target of the area under the concentration-time curve over 24 h in steady state divided by the minimum inhibitory concentration 50–100 mg⋅h/L has not yet been established. Moreover, most studies have focused on critically ill patients, yet there have been no studies in the field of renal transplantation. To optimize the dosage strategy and reduce the risk of toxicity, a population pharmacokinetics model of polymyxin B with the Phoenix NLME program was developed in our study. A total of 151 plasma samples from 50 patients were collected in the present study. Polymyxin B plasma concentrations were measured by high-performance liquid chromatography-tandem mass spectrometry. A one-compartment model adequately described the data, and the clearance and volume of distribution were 1.18 L/h and 12.09 L, respectively. A larger creatinine clearance was associated with increased clearance of polymyxin B (*p* < 0.01). Monte Carlo simulation showed that a regimen of a 75 mg loading dose with a 50 mg maintenance dose was a better option to achieve an optimal therapeutic effect (minimum inhibitory concentration ≤1 mg/L) and to reduce the incidence of side effects for patients with renal impairments. The developed model suggested that dosing adjustment should be based on renal function in renal transplant patients.

## Introduction

Infection due to multidrug-resistant (MDR) Gram-negative bacteria has become an extreme challenge, as MDR organisms have become resistant to most currently available antibiotics, resulting in limited treatment options. Polymyxin B, which was withdrawn from the market due to the high risk of neurotoxicity in the 1970s, has been reused for its high sensitivity against MDR Gram-negative bacteria (Li et al., 2006; Zavascki et al., 2007; Nation et al., 2019).

According to the results from previous pharmacokinetics and pharmacodynamics (PK/PD) studies, the bactericidal activity of polymyxin B is best correlated with the area under the concentration-time curve over 24 h in steady state divided by the minimum inhibitory concentration (AUC_0–24h_/MIC) (Mohamed et al., 2012). Moreover, the latest guidelines recommended a target plasma AUC_0–24h_ of 50–100 mg⋅h/L to achieve bactericidal activity against an isolate with an MIC of 2 mg/L (the EUCAST and CLSI breakpoints) (Tsuji et al., 2019). However, due to the lack of rigorous PK data, the optimal dosing strategies for polymyxin B remain poorly defined. Previous population PK (PPK) studies of polymyxin B showed significant individual differences across patients (Zavascki et al., 2008; Kwa et al., 2011; Sandri et al., 2013a; Sandri et al., 2013b; Avedissian et al., 2018; Kubin et al., 2018; Manchandani et al., 2018; Miglis et al., 2018; Lakota et al. 2018) found that when given the recommended dosage, only 71% of patients could achieve the AUC_0–24h_ target, which might either lead to a poor response or a higher incidence of renal injury (Lakota et al., 2018). Moreover, the significant covariates of polymyxin B pharmacokinetics are controversial. Sandri et al. found that the total body weight was correlated with the clearance rate of polymyxin B, while Miglis et al. found that patients with lower total weight might not be able to achieve a ≥90% probability of target attainment (PTA) by using weight-based regimens (Sandri et al., 2013b; Miglis et al., 2018). In addition, as polymyxin B was excreted unchanged in urine, some studies found no correlation between polymyxin B clearance (CL) and creatinine clearance (CrCL), whereas Wang et al. found that CrCL was a significant covariable for the CL of polymyxin B (Wang et al., 2020). Further studies are warranted to determine the characteristics of polymyxin B pharmacokinetics.

Due to the frequent use of antimicrobials, prolonged hospitalization and the immune-suppression state, renal transplant recipients are at high risk of MDR Gram-negative infection, which generates an ever-increasing need for polymyxin B employment in their treatment (Kaplan and Meier-Kriesche, 2004; Sayegh and Carpenter, 2004). However, no pharmacokinetics analysis of polymyxin B for patients with kidney transplant has been conducted yet, and the optimal dosage for these patients is not available. It is generally acknowledged that the pharmacokinetics of patients with solid organ transplantations are different from those of normal patients (Han et al., 2010; Chen et al., 2015; Lin et al., 2018). Therefore, the present recommended regimen might not be suitable for renal transplant patients, which might lead to decreased efficacy or a higher risk of toxicity, especially nephrotoxicity. The objectives of our study were to characterize the PPK of polymyxin B in renal transplant patients, to propose dosage regimens of polymyxin B to achieve the target plasma concentration using Monte Carlo simulation, and to investigate the toxicity of polymyxin B in renal transplant recipients.

## Materials and Methods

This prospective study was approved by the Ethics Committee of the Second Xiangya Hospital, Central South University. Informed consent was obtained from all patients or legal representatives of the patients (No. ChiCTR1900022231). Renal transplant patients (aged ≥18 years) who received intravenous polymyxin B (sulfate; Polymyxin B for Injection, China, SPH NO.1 BIOCHEMICAL and PHARMACEUTICAL CO. LTA) for ≥48 h were enrolled. Patients were excluded if they were pregnant, allergic or intolerant to polymyxin B and lacked the necessary data (weight or some renal function index).

All patients accepted at least 3 days of polymyxin B infusion, and the administration interval of polymyxin B was 12 h with an infusion duration of 60–120 min. One to six blood samples (2 mL) were randomly collected 30 min before the sixth dose of polymyxin B and at 0, 0.5, 1, 2, 4, 6, and 8 h after the end of infusion. Blood samples were centrifuged for 10 min (3,400 rpm/min), and the supernatant was immediately stored at −80°C until analysis. A validated high-performance liquid chromatography-tandem mass spectrometry (HPLC-MS/MS) was used to measure the concentrations of polymyxin B1 and polymyxin B2 (Thomas et al., 2012). Briefly, a Shim-pack GIST C18 (2.1*100 mm, 3 μm, Shimadzu) column was used. Gradient chromatography was performed with solvent A (0.1% formic acid in water) and solvent B (acetonitrile). The following gradient elution was performed at a total flow of 0.6 ml/min for analyte elution: 10–60% B from 0 to 5 min, 60%–90% B from five to 5.10 min, 90% from 5.10 to 6 min, 90%–10% B from six to 6.10 min, and 10% B from 6.10 to 10.0 min. The elution time was 10 min, and the injection volume was 2 μL. The interday precision was <12%, the intraday precision was <9%, and the accuracy ranged from 96.1 to 110.4%. The limit of quantification was 0.03 mg/L.

The following data were collected: age; sex; weight; maintenance doses; time and route of administration; site of infection; microbes; comorbidities; liver function indexes, such as alanine aminotransferase (ALT), aspartate aminotransferase (AST), total bilirubin (TBIL), direct bilirubin (DBIL), and albumin (ALB); and renal function indexes, such as uric acid (UA), blood urea nitrogen (BUN), and CrCL. CrCL was calculated according to the Cockcroft-Gault equation (Janmahasatian et al., 2005). CrCL ≤80 ml/min was defined as renal dysfunction, and when the basic CrCL decreased by more than 25%, nephrotoxicity was defined. Risk, injury, failure, loss and end-stage renal disease criteria were used to define acute kidney injury (AKI) in patients (Bellomo et al., 2004). Neurotoxicity was defined when any neurological symptoms or signs occurred during polymyxin B treatment, including dizziness, vertigo, visual disturbances, confusion, hallucinations, ataxia, seizures, and facial and peripheral paresthesia. The observed AUC_0–24h_ of patients was calculated by daily dose/CL.

### Population Pharmacokinetic Analysis

The concentration-time data of polymyxin B were analyzed with a nonlinear mixed effect modeling approach with the Phoenix NLME program (version 8.1. Pharsight, A Certara Company, United States of America). The first-order conditional estimation-extended least square method (FOCE-ELS) was used to develop the PPK model. One- and two-compartment models with linear elimination were evaluated for structure model selection based on the objective function value (OFV) and Akaike information criterion (AIC). The interindividual variability of PK parameters was described by an exponential error model. Residual variability was selected with an additive error model, proportional error model and combined error model. The covariates considered for the modeling included age, weight, ALT, AST, TBIL, DBIL, ALB, BUN, UA, and CrCL. The median of the covariate was used to normalize the covariate, and a stepwise method was used to screen the covariates. A reduction in OFVs of >3.84 (*p* < 0.05) was considered to be statistically significant for the inclusion of one additional parameter in the forward inclusion steps, and an increase in OFVs of >6.63 (*p* < 0.01) was considered to be statistically significant in the backward elimination steps.

Goodness-of-fit plots were used to assess the validity of the population PK model, which included measured concentrations (DV) versus population prediction (PRED) and individual population prediction (IPRED) plots and conditional weighted residuals (CWRES) versus time and population prediction (PRED) plots. One thousand resample data points were generated by using the bootstrap method to assess the accuracy of the population PK parameters from the final model. The 95% confidence interval (CI) of each parameter from bootstrapping should encompass the estimates of the final model, and the biases should be less than 10% with no bias across zero. Moreover, a prediction-corrected visual predictive check (pc-VPC) was used to evaluate the final model performance. A total of 1,000 replicates were calculated based on Monte Carlo simulation, and the 90% confidence intervals of the fifth, 50th, and 95th percentiles of the simulated concentrations were visually compared with the actual observed data.

### Monte Carlo Simulation

Monte Carlo simulations (n = 1,000) were conducted using metrics to determine the PTA for *f*AUC_0–24h_/MIC ≥20 at various MICs (0.5–2 mg/L) in patients with different renal functions (CrCL: 10–80 ml/min), where *f* is the unbound fraction of polymyxin B (assumed to be 0.42) (Sandri et al., 2013b). The dosages were selected according to the most commonly used regimens in renal transplant patients. The regimens were as follows with a 2 h infusion: a single 50 mg loading dose followed by either 30 mg every 12 h or 40 mg every 12 h, either a loading dose of 75 mg or 100 mg followed by 50 mg every 12 h, and a 150 mg loading dose followed by 75 mg every 12 h.

AUCs were calculated for each simulated dosing regimen and each group of renal functions (CrCL≤30 ml/min, 50 ml/min, and 80 ml/min) after 24 or 72 h of therapy to evaluate the probabilities of efficacy and toxicity, respectively. An AUC_0–24h_ of 50–100 mg·h/L was taken as the efficacy exposure of interest according to previous studies (Tsuji et al., 2019).

## Results

### Demographic Data

In total, we collected 151 plasma samples from 50 patients with renal transplant for PK analysis of polymyxin B. The demographic characteristics of the patients are summarized in Table 1. The range of CrCL was 4.29–90.7 ml/min, and most patients (92.0%) were diagnosed with renal insufficiency, of which 29 patients had CrCL less than 30 ml/min. Eleven patients had continuous renal replacement therapy (CRRT) during the polymyxin B treatment, and the hemodialysis time was 4 h. Only two patients used loading doses, and the most commonly used maintenance doses were 40 mg and 50 mg q 12 h.

**TABLE 1:** Patients’ demographic characteristics.

Characteristic	Value^a^
Age (year)	43.5 (18–66)
Female/Male	18/32
Actual body weight (kg)	57.8 ± 12.4
Polymyxin B duration (day)	9 (5–52)
Polymyxin B maintenance dose	
50 mg q 12 h	20 (40.0%)
40 mg q 12 h	22 (44.0%)
Other doses	8 (16.0%)
Continuous renal replacement	11 (22.0%)
Renal dysfunction	46 (92.0%)
Diabetes mellitus	7 (14.0%)
Hypertension	28 (56.0%)
UA (µmol/L)	363.0 (94.8–752.6)
ALB (g/L)	34.7 (22.7–49.0)
BUN (mmol/L)	20.2 (2.65–61.9)
CrCL (ml/min)	22.2 (4.29–90.7)
ALT (U/L)	11.7 (4.3–226.7)
TBIL (µmol/L)	6.8 (2.6–30.6)
AST (U/L)	15.7 (9.8–76.7)
DBIL (µmol/L)	2.8 (1.3–10.3)
Site of infection	
Pulmonary	45 (90.0%)
urinary tract	4 (8.0%)
bloodstream	1 (2.0%)
Microbe	
P. aeruginosa	1 (2.0%)
Klebsiella pneumoniae	3 (6.0%)
Escherichia coli	3 (6.0%)
None	43 (86.0%)

a Values are the median (range), mean ± SD or number (%); UA, uric acid; ALB, albumin; BUN, blood urea nitrogen; CrCL, creatinine clearance; ALT, alanine aminotransferase; TBIL, total bilirubin; AST, aspartate aminotransferase; DBIL, direct bilirubin.

Table 2 described how many times of blood samples were taken from each patient, and most of the samples were taken from 39 patients at three different times. Overall, 49 (32.5%) samples were collected at 0.5 h before the sixth infusion, and 48 (31.8%) were collected at 0 h after, the rest were taken at random times during this infusion interval. The plasma concentrations of polymyxin B ranged from 0.44 mg/L to 8.15 mg/L, and the mean AUC_0–24h_ was 74.60 ± 17.81 mg·h/L.

**TABLE 2:** Distribution of blood samples.

Number of patients	Blood samples taken at different times from each patient
2	1
3	2
39	3
5	4
1	6

### Development of the PPK Model

A one-compartment PK model with first-order elimination fit the best in describing the data. The development and selection of the basic model is shown in Table 3. A proportional error model was used to evaluate the residual variability. CrCL was the only significantly effective covariate for the CL of polymyxin B. No covariate was statistically significant for the volume of distribution (V). The final PK model equations were as follows: CL (L/h) = 1.18*(CrCL/22.2)^^0.14^*exp (ηCL); V (L) = 12.09*exp (ηV). The PPK estimate parameters are presented in Table 4.

**TABLE 3:** Model selection and development.

Model description		OFV^a^	ΔOFV^b^	AIC^c^
One-compartment mode		465.41	—	471.41
Two-compartment model		465.41	—	475.41
The choice of residual variability model (One-compartment model)				
Addictive error model		402.93	—	412.93
Proportional error model		375.14	—	385.14
Combined error model		375.15	—	387.14
Full covariate model		363.90	11.24	375.90

a objective function value.

b change in objective function value in the covariate model.

c Akaike information criterion.

**TABLE 4:** Population PK parameter estimates in the final model and bootstrap.

Parameter (unit)	Final model results		Bootstrap results	
	Estimate (shrinkage%)	%CV^a^	Median	95% CI^b^
V (L)	12.09	6.52	11.98	10.71–13.67
CL (L/h)	1.18	4.15	1.17	1.08–1.27
*Θ*_CrCL_	0.14	29.35	0.14	0.05–0.21
Interindividual variability				
*ω* ^2^_V_	0.04 (40.70)	20.74	0.04	—
*ω* ^2^_CL_	0.06 (7.16)	24.49	0.06	—
Residual variability				
*σ*	0.17	—	0.17	0.13–0.21

a coefficient of variation.

b 2.5^th^ and 97.5^th^ percentile of the ranked bootstrap parameter estimate; *Θ* exponent for CrCL as a covariate for CL; *ω* interindividual variation; *σ* intraindividual variation.

**FIGURE 1:**
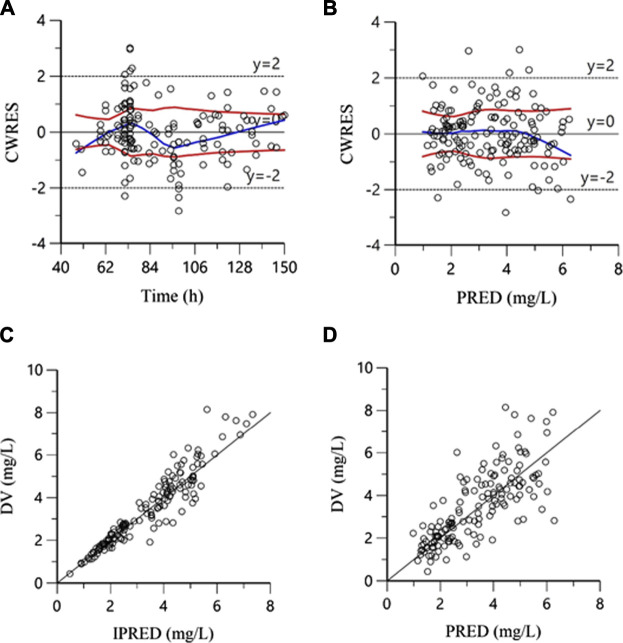
Goodness-of-fit plots for the final model. **(A)**: Conditional weighted residuals versus time; **(B)**: Conditional weighted residuals versus population predicted concentrations; **(C)**: Measured concentrations (DV) versus individual predicted concentrations; **(D)**: Measured concentrations (DV) versus population predicted concentrations.

The goodness-of-fit plots in the final model are shown in Figure 1. The plots of DV versus IPRED and PRED indicated no structural bias. The plots of CWRES versus PRED and time showed a random distribution around zero, and most of the plasma concentrations were scattered within −2 to 2, showing that the structure of the final model was not biased and that the model was acceptable.

**FIGURE 2:**
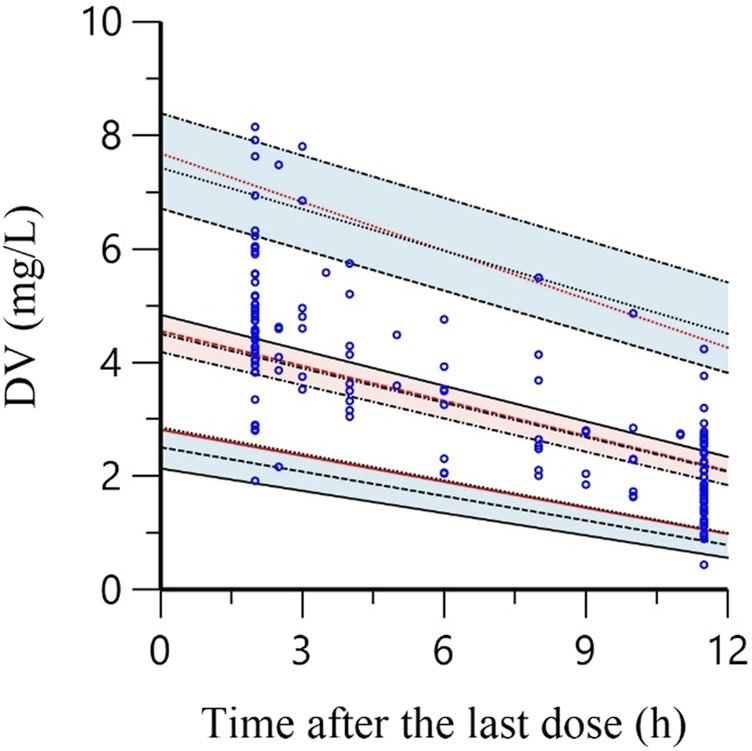
The prediction corrected-visual predictive check of the final model. Blue circles represent the observed polymyxin B concentrations. Solid red lines indicate the 5th, 50th and 95th percentiles of the observed data. Black dotted lines show the 5th, 50th and 95th percentiles of the simulated data. The 3 shaded areas represent the 90% confidence intervals of the 5th, 50th and 95th percentiles of the simulated concentrations.

The estimates of the bootstrap method are presented in Table 4. The estimates of the final model were similar to the median estimates of the bootstrap, and they were within the 95% CI from the bootstrap analysis, which did not include zero. Additionally, their deviation was less than 10%, indicating that the parameters of the final model were accurate. The result of pc-VPC is shown in Figure 2. Most concentrations were within the 90% CIs, indicating that the final model had a good description of the original data.

### Monte Carlo Simulation

In the analysis of PTA, all regimens aside from a 50 mg loading dose with 40 mg every 12 h achieved the target concentration at MIC≤ 1 mg/L in all renal function groups on day 3. Most of the simulated regimens could not achieve adequate target attainment at MICs at the current CLSI and EUCAST breakpoint of 2 mg/L, except 150 mg loading dose with 75 mg maintenance dose in patients with CrCL≤10 ml/min (Table 5).

**TABLE 5:** PTA based on different CrCL and doses administration on day 3.

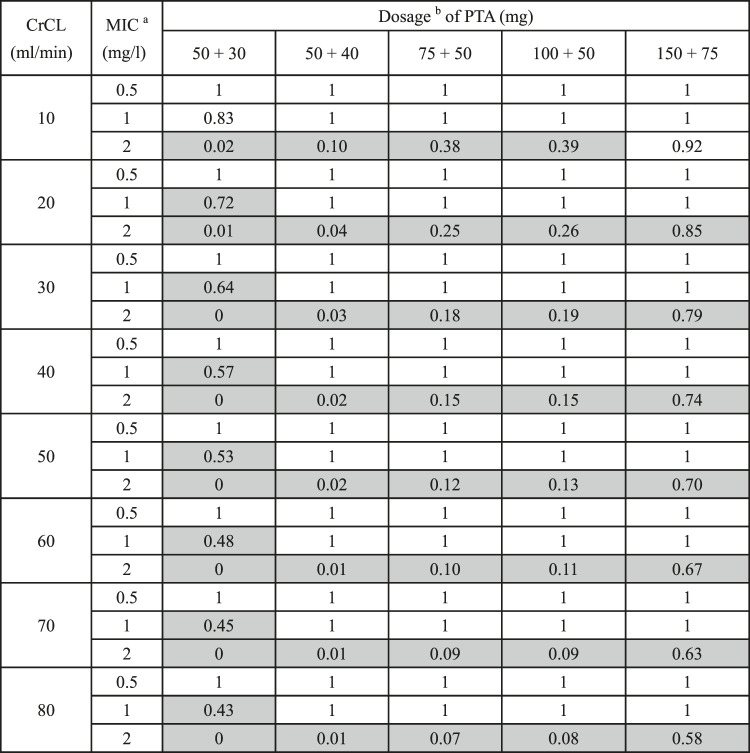

a minimum inhibitory concentration.

b loading dose + maintenance dose twice daily; CrCL, creatinine clearance; MIC: minimum inhibitory concentration; PTA, probability of target attainment; gray background: groups that have not reached the target PTA%.

The simulated results of AUC_0–24h_ at day 1 and day 3 are shown in Table 6. All loading doses made it possible to obtain steady concentrations on day 1, which was in accordance with the guidelines that loading doses were essential for polymyxin B treatments. However, loading regimens still failed to produce probabilities of efficacious exposures at MICs ≥2 mg/L. A single loading dose of 75 mg and a 50 mg maintenance dose was sufficient to achieve the target concentrations on day 1 or day 3 in approximately 90% of patients.

**TABLE 6:** AUC_0–24h_ based on different CrCL and doses administration on day 1 and day 3.

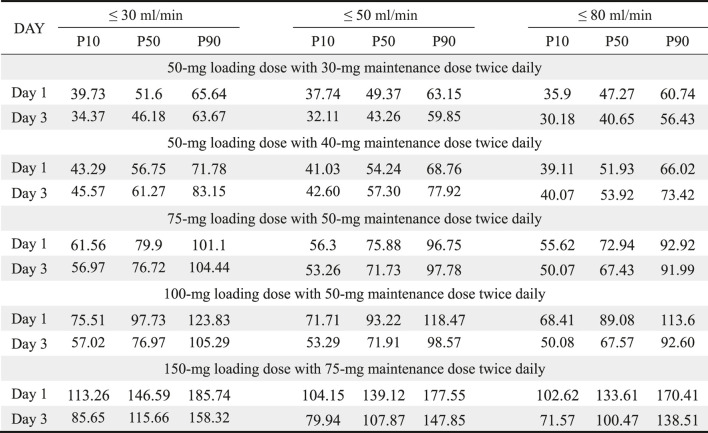

AUC_0–24h_: area under the concentration-time curve over 24 h; CrCL: creatinine clearance; P10, 10^th^ percentile; P50, 50^th^ percentile; P90, 90^th^ percentile.

### Adverse Effects

A total of five patients experienced nephrotoxicity according to our definition (3–11 days), four of whom were classified as risk (3–7 days), and one as injury (11 days). However, different degrees of neurotoxicity were noted in 31 patients (62.0%): 29 had facial and peripheral paresthesia, three had seizures, and three had dizziness. Seventeen patients (34.0%) had dermatitis. However, those neurotoxicity symptoms disappeared after polymyxin B withdrawal. Seven patients had pigmentation, and all disappeared 1 month after polymyxin B withdrawal.

## Discussion

This study developed a PPK model of polymyxin B for Chinese renal transplant patients. To the best of our knowledge, this is the first study that investigated the pharmacokinetic features and optimized dosage of polymyxin B in renal transplant patients.

In the present study, a one-compartment model was used to describe the original data, which was consistent with some of the previous studies (Kubin et al., 2018; Manchandani et al., 2018). The estimated median value of clearance, CL (1.18 L/h), was lower than the value reported in the previous one-compartment models (CL: 2.37–2.5 L/h) (Kubin et al., 2018; Manchandani et al., 2018). This difference might be mainly due to impaired renal function in the renal transplant patients in this study (CrCL: 20.89 ml/min, range: 4.29–78.84 ml/min) because we identified CrCL as a significant predictor of CL in the covariate analysis. After including the covariate of CrCL in the model, the interindividual variability of CL was reduced from 28.11 to 24.49% in the final PPK model. It has been debated whether CrCL has a significant effect on the CL of polymyxin B. Sandri et al. found that polymyxin B is not significantly eliminated by the kidneys, and some other PPK studies demonstrated that polymyxin B clearance does not depend on CrCL (Sandri et al., 2013b; Kubin et al., 2018; Miglis et al., 2018). According to these results, no dose adjustment was recommended by the international consensus guidelines (Tsuji et al., 2019). However, urinary recovery varied considerably between different patients in a previous study (range, 0.98–17.4%), which indicated that the renal excretion of polymyxin B in some patients might not be very low (Sandri et al., 2013b). Moreover, Manchandani et al. found that CrCL was a significant covariate for CL of polymyxin B, and the CL value in renal dysfunction patients was found to be lower than (marginal significance) that in patients without renal impairment according to Thamlikitkul’s results (Thamlikitkul et al., 2017; Manchandani et al., 2018). With larger samples and more severe renal impairment, the influence of CrCL on polymyxin B clearance seemed to be more significant in our study, but larger PK studies in patients with renal insufficiency are needed to validate the relationship between renal function and polymyxin B clearance.

We also assessed the relationship between total body weight and polymyxin B CL. Similar to other studies, a general lack of a significant linear relationship between weight and polymyxin B clearance was found in the renal transplant patient population (Kubin et al., 2018; Miglis et al., 2018; Wang et al., 2020). In addition, a previous Monte Carlo simulation found that in the weight-based dosing scheme, patients in the lowest body weight strata were far from achieving >90% PTA, while patients in the highest body weight strata had a higher incidence of toxic exposures (Miglis et al., 2018). Future work should quantify the full relationship between weight and polymyxin B clearance and whether any one weight-based dosing scheme should be used.

The mean V (12.09 L) in our study was also lower than the data reported by previous studies (V: 34.3–34.4 L) (Kubin et al., 2018; Manchandani et al., 2018), which indicates that polymyxin B might have less tissue distribution in renal transplant patients. Polymyxin B is mainly distributed in the kidney, and the renal tissue/serum concentration ratio of polymyxin B was 19.62 at 6 h after dosing in rats (Manchandani et al., 2015). Therefore, the variation in polymyxin B accumulation in the kidney might be responsible for the differences in the apparent volume of distribution. In addition to the exceptional pharmacokinetic characteristics of renal transplant patients, most of our patients had severe renal impairment. Therefore, decreased glomerular filtration might reduce the distribution of polymyxin B in the kidney in these patients, which leads to a lower V value. This speculation that patients with renal impairment might have lower renal exposure to polymyxin B is also consistent with a previous study, which found that when both groups were given high-dose polymyxin B, patients with CrCL ≥90 ml/min were more likely to develop AKI than patients with mild renal impairment (John et al., 2017). These results highlighted the need for further investigation to better define the distribution of polymyxin B.

This is the first study to simulate the PTA and AUC_0–24h_ of different regimens in patients based on renal function. Our results showed that the regimen of a 50-mg loading dose with a 40-mg maintenance dose was sufficient to reach the target PTA with an MIC ≤1 mg/L for patients with renal dysfunction (CrCL≤ 80 ml/min), which was much lower than the dosage recommended by other studies (Miglis et al., 2018; Tsuji et al., 2019). This might be due to the unique PPK features of polymyxin B in renal transplant patients, which was in accordance with previous studies. Xiao-bin et al. found that the voriconazole CL in patients with renal transplant was similar to the result of patients with lung transplant, which was much lower than that in normal patients (Lin et al., 2018). However, most of the simulated regimens failed to achieve adequate target attainment at an MIC of 2 mg/L, which suggests that patients might experience poor clinical outcome when treated with polymyxin B in such circumstances.

AUC_0–24h_ of regimens of ≥50 mg maintenance dose all achieved the proposed therapeutic target of 50–100 mg·h/L on day 3, and with a loading dose of 100 mg, the target concentration can be quickly achieved on the first day, which is more prominent for critically ill patients. However, polymyxin B seemed tolerated poorly in the renal transplant patients due to the neurotoxicity, thus, the determination of optimal dosing strategies should be based on the balance between the toxicity and the need for early efficacious exposures.

It is well known that nephrotoxicity is the major adverse effect of polymyxin B, with a wide range from 26.9 to 60% (Plachouras et al., 2009; Akajagbor et al., 2013; Dubrovskaya et al., 2015; Rigatto et al., 2015). In the present study, however, the incidence of AKI was extremely low, with only five patients showing nephrotoxicity. As polymyxin B-induced nephrotoxicity is a dose-limiting adverse effect, our result seemed to be consistent with the previous finding that patients with renal impairment might have a lower incidence of AKI due to the decreased percentage of tubular reabsorption with CrCL and thus the lower renal exposure to polymyxin B. In addition, older age has been found to be a risk factor for polymyxin B-induced AKI, and the lower risk of AKI in our patients might also be due to the younger average age (Rigatto et al., 2015).

The incidence of neurotoxicity was much higher than noted before (62.0 vs 7%) and has become the dose-limiting factor in our study (Arnold et al., 2007). The mechanism of polymyxin B-induced neurotoxicity is not yet clear, and some studies have demonstrated that colistin-induced neurotoxicity is dose-dependent and significantly associated with the apoptosis of neuronal cells (Lu et al., 2017a; Lu et al., 2017b; Dai et al., 2017; Dai et al., 2018a; Dai et al., 2018b). Considering their similar structures, this mechanism might also fit polymyxin B; therefore, the higher exposure of polymyxin B in renal transplant patients might induce more severe neurotoxicity. In addition, the immunosuppressive agents taken by our patients, such as tacrolimus and cyclosporine, can also induce neurotoxicity (Eidelman et al., 1991; Wijdicks et al., 1994; Erer et al., 1996), and this combination might increase the incidence of this adverse effect.

There are several limitations in the present study. First, the sample size was small, which resulted in the limited analysis of risk factors for neurotoxicity. Second, all of our patients had renal dysfunction, and we could not investigate the PKs of renal transplant patients with normal renal function.

To our knowledge, this is the first population PK model and CrCL-based simulation established in renal transplant patients. Our results suggested that renal function might have a significant effect on the clearance of polymyxin B, and an adjusted dosage regime might be needed in patients with renal impairments. Further investigation of the relationships between polymyxin B pharmacokinetics and renal function is urgently needed.

## Data Availability

The original contributions presented in the study are included in the article/supplementary material, further inquiries can be directed to the corresponding authors.
